# The working alliance inventory – short version: psychometric properties of the patient and therapist form in youth mental health and addiction care

**DOI:** 10.1186/s40359-024-01754-1

**Published:** 2024-05-31

**Authors:** Patty van Benthem, R. M. van der Lans, A. Lamers, P. Blanken, R. Spijkerman, R. R.J.M. Vermeiren, V. M. Hendriks

**Affiliations:** 1 Parnassia Addiction Research Centre (PARC), Brijder Addiction Care, Zoutkeetsingel 40, The Hague, 2512 HN The Netherlands; 2 LUMC Curium, Leiden University Medical Center, Leiden, the Netherlands; 3 University of Amsterdam, Amsterdam, the Netherlands; 4 Opvoedpoli, Amsterdam, the Netherlands

**Keywords:** The working alliance inventory-short version, Youth treatment, Youth mental health care, Youth addiction care, Multilevel confirmatory factor analyses, Hierarchical alliance data, Psychometric properties, Therapeutic alliance, Patient form, Patient form, Therapist form

## Abstract

The therapeutic alliance is considered to play an important role in youth treatment. The commonly used versions of the Working Alliance Inventory (WAI) are based on Bordin’s three-dimensional alliance model. However, previous psychometric studies of the WAI did not find this three-dimensional structure in youth psychotherapy. These earlier findings may indicate different perceptions of the alliance by adolescent versus adult patients, but may also be due to methodological shortcomings. The current study aims to address previous study limitations by evaluating the factor structure of the short version of the WAI (WAI-S) in youth treatment in multilevel analysis to address the hierarchical structure of the alliance data. We examined the psychometric properties of the patient (*n* = 203) and therapist (*n* = 62) versions of the WAI-S in youth mental health and addiction care and tested four multilevel models of alliance at start of treatment and 2-month follow-up. Our results suggests a two-factor model for youth and a three-dimensional model for their therapist at both time points. Since this is the first study that finds a best fit for a two-dimensional construct of alliance in youth, more research is needed to clarify whether the differences in alliance dimensions are due to measurement differences between the WAI-S for youth and therapists or whether youth and their therapists truly differ in their perceptions of the concept of alliance.

**Supplementary Information:**

The online version contains supplementary material (Appendix) available at 10.1186/s40359-024-01754-1.

## Background

In both adult and youth psychotherapy, the therapeutic alliance is considered to play an important role in therapeutic progress and treatment success. Recent meta-analyses among adults and youths found consistent, albeit moderate, associations between a strong therapeutic alliance and beneficial outcomes in mental health treatment [1–3]. The Working Alliance Inventory (WAI) [4] is the most extensively used questionnaire to assess therapeutic alliance [5], and has been adapted in more than 30 versions, including a version for patients, therapists, treatment teams, parents and observers. Specifically, more than two-thirds of the studies in the meta-analysis by Flückiger et al. [1] on adult psychotherapy used a version or adaptation of the WAI and in both meta-analyses on youth psychotherapy [2, 3] the WAI was also the most frequently used measure.

The WAI and its short forms, the WAI-Short (WAI-S) [6] and WAI-Short Revised (WAI-SR) [7] are based on Bordin’s [8] conceptual model of the therapeutic alliance or ‘working alliance’. Bordin [8, 9] describes the working alliance as the collaborative relationship between patient and therapist based on purposeful collaboration and an affective bond. Elements of the working alliance are the extent to which the patient-therapist dyad engages in purposeful collaboration, including the explicit negotiation of therapeutic tasks and goals, as well as the development of a sufficiently strong affective bond to engage in the therapeutic work during different treatment phases. In line with Bordin’s conceptualization, the working alliance is operationalized as a three-dimensional construct which incorporates the level of task collaboration (task), the degree of agreement on the treatment goals (goal) and the affective quality of the patient-therapist relationship (bond). Both the WAI and WAI-S(R) are based on this three-dimensional structure with items that fall into a total scale and three subscales, i.e. task, goal and bond.

In adult populations the WAI has shown good internal, inter-rater and test-retest reliability indices [10] but empirical evidence for its underlying three-dimensional factor structure has been mixed. Some studies confirmed the 3-factor structure [4, 6, 7, 11, 12] whereas other studies demonstrated a 2- or single factor structure [13–16] or a bi-factor hierarchical structure [6, 17]. Moreover, inter-correlations between the three subscales were high, especially the task and goal scale were highly correlated. More moderate correlations between the task and goal scales and improved model fit were found for the WAI-SR, the revised version of the WAI-S, in which negatively worded items were converted to positively worded items [7].

Distinguishing these different components of alliance has implications for research and practice. Some alliance studies focus primarily on the different components of alliance to determine which component has the highest priority at which stage of treatment [15, 16], while others see the value of alliance primarily as synergy of the different dimensions [4]. According to Bordin [8], the demands placed on the different alliance dimensions differ by type of treatment. For example, cognitive behavioral therapy may focus more on collaboration [8, 15, 18–20] while interpersonal and humanistic therapies might place a greater emphasis on the bond [8, 15, 19]. Although it is plausible that different types of treatments require different alliance `profiles`, most psychometric studies of the WAI do not differentiate between the specific alliance dimensions, especially in youth psychotherapy.

Although the WAI was originally designed for adult treatment and then adapted for use with children and adolescents [21, 22], its factor structure has not received much attention in the youth alliance literature. More research on this topic is particularly important since youth in psychotherapy may have a different understanding of the therapeutic alliance than adults. Some researchers propose that youth may view the alliance more as an affective instead of a cognitive construct [23]. Others suggest that young people do not yet fully possess the cognitive skills needed to reflect on their own behavior and emotions, to formulate long-term treatment goals, to convert these abstract goals into specific tasks and to distinguish between treatment goals and tasks [21, 24, 25]. Furthermore, it can be difficult to agree on treatment goals with young people who are often referred to treatment because of concerned parents or significant others, when they themselves may not recognize the symptoms of mental illness [26]. Finally, typical characteristics of adolescents’ developmental stage, such as the strong aspiration for independence and self-determination, may affect the formation of an affective and collaborative relationship between adolescents and their therapist [27, 28]. In sum, due to (developmental) differences, youth may experience the therapeutic alliance differently compared to adults. This may imply that Bordin’s three-dimensional alliance model and the related factor structure of the WAI do not adequately reflect alliance in youth.

The few studies that have examined the factor structure of versions of the WAI questionnaire in youth have shown inconsistent findings and do not allow definite conclusions about the dimensional structure of the WAI in youth. The original WAI [4], with 36 items for both the patient and therapist version, was first investigated by DiGiuseppe et al. [21] among youth in psychotherapy. Their factor analysis yielded one single alliance factor for youth while for therapists, besides the one single alliance factor, the three factors for goal, task and bond were found. In addition, Diamond et al. [29] found a single alliance factor structure for both the therapist- and youth-rated WAI among patients in cannabis youth treatment [29].

The 12-item WAI-S [6], a shorter version of the WAI, was investigated in two youth studies showing a single factor solution for the patient version in youths receiving treatment for anxiety [30] or depression [31]. Although findings from Cirasola et al. [31] also provide some support for a two-factor alliance structure with collaboration (goal and task combined) and bond, the correlation between these two factors was very high (*r* = 0.91). Regarding the therapist version of the WAI-S, Cirasola et al. [31] demonstrated the best fit for the two-factor model and some evidence for the three-factor model. Unfortunately, factor analytic studies on the WAI-SR are lacking in youth.

Overall, prior studies on the factor structure of the WAI and WAI-S in youth treatment provide most empirical support for the general one-factor alliance model for the patient version, while for the therapist version the findings are inconclusive. These results may indicate that the alliance structure is different in youth than in adults, but may also be explained by methodological limitations and the type of questionnaire that was investigated. A major limitation of previous studies is the lack of attention to the hierarchical ordering of the data, which may have led to biased parameter estimates, incorrectly and usually overestimated standard errors, and incorrect model fitting [32–36]. Therefore, the use of multilevel confirmatory factor analyses (MCFA) to account for hierarchical data would be more appropriate for alliance data than the traditional confirmatory analyses.

Another explanation for previous findings may be the version of the WAI that was investigated. Both the WAI and WAI-S contained two negatively worded items that could result in response bias among youth, a method effect found by Cirasola et al. [31].

In sum, therapeutic alliance is considered to play an important role in youth treatment and the emphasis on the three different alliance dimensions may vary by treatment type. To date, most psychometric studies that examined the factor structure of the WAI did not distinguish the three alliance dimensions but provided strongest support for a one-dimensional structure. These findings may indicate that the true alliance structure in youth consists of one general alliance dimension but may also be explained by methodological shortcomings, since most studies did not account for the clustering of the alliance data and examined WAI versions with negatively worded items which may lead to response bias.

In the present study we aim to address previous methodological limitations of psychometric studies on the WAI in youth by (1) testing the factor structure of the WAI-S, in which the two negatively worded items were rephrased into positively worded items, to prevent response bias, and (2) by applying multilevel confirmatory factor analysis to account for clustered data. The main objective of the present study is to examine the psychometric properties of the WAI-S in youth mental health and addiction treatment by (1) investigating whether the three-dimensional (task, goal and bond), or a one-, two- or bi-factor (level one: general alliance; level two: task, goal and bond) model of alliance adequately fits the construct of alliance in youth treatment; (2) examining whether the alliance structure is robust (a) across youths and therapists and (b) across time; and (3) taking into account the nested data structure, in which youth are nested in therapists.

## Methods

### Participants

The setting for this study is the Professional Alliance with Clients in Treatment (PACT) study – a multi-site prospective naturalistic clinical cohort study assessing the effects of therapeutic alliance and client feedback in outpatient youth mental health and addiction care. More details about the PACT study are reported in van Benthem et al. [37]. The present study included 203 youth (aged between 13 and 23 years) and 62 therapists with at least one assessment of alliance completed by the youth or therapist at the start of the treatment. The therapists’ caseloads ranged from one till 16 youth (Median = 4, Mode = 2). At two-months follow-up 183 youth were still in treatment and treated by 56 therapists. Specifically, the youth-rated WAI-S dataset consisted of 203 youth-rated WAI-S questionnaires at first-session and 183 at two-months follow-up. The therapist-rated WAI-S dataset consisted of 203 therapist-rated WAI-S questionnaires at first session and 188 at two-months follow-up.

### Instruments

Information on age, gender and demographic background was collected at the time of the first treatment session. Therapeutic alliance was assessed immediately following the first treatment session and at two-months follow-up, with the patient and therapist form of the WAI-S translated into Dutch language [38], and, as noted before in this article, with the negatively worded items rephrased into positively worded items. The WAI-S consist of 12 items, which reflect Bordin’s three-dimensional conceptualization of alliance: Task, Goal and Bond (4 items each). Youths and therapists were required to rate each item on a 5-point Likert scale ranging from ‘never’ (1) to ‘always’ (5) with higher scores indicating better quality of the alliance. Data were collected by independent and trained research assistants.

### Statistical analyses

A series of multilevel confirmatory factor analysis (MCFA) was conducted to examine the factor structure of the youths’ and therapists’ ratings of the WAI-S. Because in our study the youths were nested within therapists, we conducted multilevel CFA to estimate both within- and between therapist variances. In addition, to address the categorical nature of the alliance data, as all items were rated on 5-point Likert scales, the mean and variance adjusted weighted least squares (WLSMV) estimator was used. Four alliance models were tested and compared with each other: (1) one-factor model with all items loading into a general alliance factor; (2) two-factor model with both the task and goal items loading into one, combined collaboration factor and the bond items on the other, second factor; (3) three-factor model with task, goal and bond; (4) bi-level model with general alliance on level one and task, goal and bond on level two. To test the robustness of our model, across alliance rater and across time, each MCFA was run separately for youths’ and therapists’ alliance ratings and for each time point.

We evaluated the model fit of all the MCFA models, i.e. the discrepancy between model predictions and the observed data, by assessing the fit indices: Root Mean Square Error of Approximation (RMSEA) < 0.08, the Comparative Fit Index (CFI) > 0.90, the Standardized Root Mean Square Residual (SRMR) < 0.08 and the Tucker-Lewis Index (TLI) > 0.95 [39–43]. In order to improve model fit we explored the modification indices (MIs) of the best fitting model. Potential (MIs at least 2 times larger than other MIs were taken into account) dual loadings and residual correlation parameter constraints were freed, MIs were re-estimated and this process was repeated until the MI suggested no further improvements of the model fit. In each sample the whole cycle of MIs was used.

After finding the best fitting model of the WAI-S youth and WAI-S therapist at first session we evaluated measurement invariance. We tested two hierarchical levels of measurement invariance, i.e. configural and scalar. Due to the categorical nature of our data metric invariance was not tested [44]. Configural invariance refers to the same factor structure across groups. When the factor structure of the best fitting model was found across samples, with at least adequate fit, we considered this as an indication of configural invariance. Next, we added the invariance constraints of the scalar invariance model with both factor loadings and item intercepts constrained to be equivalent across groups. Subsequently, change in model fit was evaluated by Chen’s [45] criteria: differences in CFI (∆ < 0.01), RMSEA (∆ < 0.015), SRMR (∆ < 0.02) and TLI (∆ < 0.01). Cronbach’s alpha (0.70 to 0.79 acceptable; 0.80 to 0.89 good; 0.90 to 1.00 excellent) [46] was computed to analyze internal consistency.

The degree of nesting due to youths being treated by the same therapist was assessed with intraclass correlations (ICC’s), because in ‘real life’ cross-sectional studies the ICC is no higher than 0.20 [47, 48], we considered ICCs of 0.10–0.20 as moderate and > 0.20 as high. Missing data were assumed to be missing completely at random and were handled using listwise deletion, given that significant differences in baseline mental health problems, substance use problems, and therapeutic alliance between treatment completers and non-completers were not found (see Table S1 in the Appendix). Data processing, descriptive analyses, internal consistency and intraclass correlations were conducted with IBM SPSS Statistics for Windows, version 25.0 (IBM Corp., Armonk, N.Y., USA). Multilevel confirmatory factor analysis (MCFA) and measurement invariance assessment was conducted in Mplus version 8.8, respectively [49].

## Results

### Descriptive statistics

Demographic information for patients and therapists at first session is displayed in Table 1.

**Table 1:** Demographic background for the first session WAI-S patient and therapist samples

	WAI-S patient sample (*n* = 203) %/mean(s.d.) median	WAI-S therapist sample (*n* = 62) %/mean(s.d.) median
**Demographic background**		
Age (years)	18.2 (2.6) **18.0**	38.2 (8.8) **36.5**
Male (%)	49.8	21.0
Cultural background Non-Dutch (%)	25.1	10.0

Table 2 shows means and standard deviations for each WAI-S item in both samples at first session (for two-months follow-up, see the supplementary Table S2 in the Appendix). Internal consistency of the WAI-S was overall acceptable to good among patients and therapists (Table 3). Correlations of WAI-S factors between patient and therapist version were null or negligible at first session and significant at 2-month follow-up (shown in the supplementary Table S3 in the Appendix). Descriptive statistics and correlations matrix of WAI-S in both samples at both time points can be found in the supplementary Tables S5–S10 in the Appendix. The three-factor model could not be reliably estimated in the youth sample at both first session and 2-month follow-up. The bi-factor model failed in both the youth and therapist sample and at both time points due to identification problems. The three-factor model in the youth sample and the bi-factor model are not reported.

**Table 2:** Means and standard deviations of the first session WAI-S items patient and therapist version

		mean(s.d.)		mean(s.d.)
WAI-S	Patient version		Therapist version	
**Task**				
**Item 2**	What I do in this treatment gives me more insight into my problems	3.4 (1.1)	The youth and I have confidence in the usefulness or our current activities in the treatment	3.6 (0.8)
**Item 8**	The therapist and I agree about what is important for me to work on	4.2 (0.8)	The youth and I agree about what is important to work on	3.8 (0.7)
**Item 10**	I think that my contribution to this treatment will help me to achieve the changes I want	3.8 (1.0)	I am confident that the things we do in treatment will help the youth to achieve the changes he/she wanted	3.9 (0.7)
**Item 12**	I believe that the way we work on the problems is the right way	3.7 (1.0)	The youth believe(s) that the way we work on his/her problems is the right way	3.4 (0.8)
**Goal**				
**Item 1**	One result of this treatment is that it is clearer for me how I can change	3.3 (1.0)	One result of this treatment is that it is clearer for the youth how he/she can change	3.8 (0.7)
**Item 4**	The therapist and I work together in determining the treatment goals	4.2 (0.9)	The youth and I worked together to determine treatment goals	4.0 (0.9)
**Item 6**	The therapist and I work on treatment goals we both agreed upon	4.4 (0.8)	The youth and I work on treatment goals we both agreed upon	4.2 (0.8)
**Item 11**	The therapist and I have formed a clear understanding of the kind of changes that would be good for me	3.8 (1.0)	The youth and I have formed a good understanding of the kind of changes that would be good for him/her	3.7 (0.8)
**Bond**				
**Item 3**	I believe the therapist likes me	4.1 (0.9)	I believe that the youth likes me	3.6 (0.8)
**Item 5**	The therapist and I respect each other	4.7 (0.5)	The youth and I respect each other	4.4 (0.7)
**Item 7**	I feel appreciated by the therapist	4.3 (0.9)	I appreciate the youth as a person	4.6 (0.6)
**Item 9**	I feel the therapist cares for me, even if I do things he/she disapproves of	3.6 (1.1)	I respect the youth, even if he/she does things I don’t approve of	4.4 (0.7)

**Table 3:** Cronbach’s alpha’s of the first session WAI-S patient and therapist version

	WAI-S patient version (*n* = 203)						WAI-S therapist version (*n* = 62)				
	Task	Goal	Bond	Collaboration	Total		Task	Goal	Bond	Collaboration	Total
**Cronbach’s alpha value**	0.73	0.71	0.76	0.84	0.88		0.81	0.75	0.81	0.87	0.89

**Factor structure WAI-S youth.** The fit statistics of the three models tested for the WAI-S patients at both measurement times are presented in the upper part of Table 4.

**First session.** As can be seen from Table 4, the one-factor model did not fit well in terms of the RMSEA (0.130) and TLI (0.899) criteria, whereas an acceptable fit was suggested by the CFI (0.918) and SRMR (0.079). Also, the two-factor model did not fit well according to the RMSEA (0.112) and TLI (0.940), while the CFI (0.940) and SRMR (0.068) criteria suggested an acceptable fit. The three-factor model had a correlation greater than 1.

(*r* = 1.04) between the scales Task and Goal, an indication that the model does not fit the data in a meaningful way [50]. Hence, the three-factor model output was inadmissible and is not reported. Inspection of the MIs of the two-factor model showed that the residual correlation between item 1 (Goal factor; “clearer how to change”) and item 2 (Task factor; “insight in problems”) had an MI of 46.09 which was more than 3 times greater than the other MIs. As seen in Table 4, the modified two-factor model had acceptable goodness-of-fit assessed by the CFI (0.958) and SRMR (0.060), while the TLI (0.947) and RMSEA (0.094) suggested a poor fit. To further improve model fit, we inspected the MIs for the modified two-factor model and found that allowing item 6 (Goal factor; “treatment goals both agreed upon”) to load on the Bond factor significantly improved fit (MI = 24.3). We therefore decided to allow item 6 to load on both the Goal and Bond factor. This modified two-factor model had acceptable goodness-of-fit assessed by the CFI (0.966), SRMR (0.055) and the TLI (0.956) only the RMSEA (0.086) suggested a poor fit. To further improve model fit, we inspected the MIs for the modified two-factor model and found one suggestion to improve model fit. Allowing item 4 (Goal; ‘work together in determining treatment goals’) to load also on the Bond factor would improve the two-factor modified model’s fit with MI 30.87. We re-ran the modified two-factor model, including one residual correlation and two dual-loadings and found a good goodness-of-fit given the absolute indices CFI (0.979), RMSEA (0.068), SRMR (0.048) and the TLI (0.972) (Table 4). The Bond and Collaboration factors in the modified two-factor model showed a latent variable correlation of *r* = 0.62. A visual representation of the WAI-S youth modified two-factor model at first session is shown in figure S1 in the Appendix.

**Two-months.** Consistent with the first session WAI-S models, the one- and two-factor models did not fit well according to the RMSEA (0.116 and 0.111 respectively) and TLI (0.941 and 0.947), whereas the CFI (0.952 and 0.957) and SRMR (0.071 and 0.069) suggested an acceptable fit for both models (Table 4). Again, the three-factor model was inadmissible with a correlation above 1 between the Task and Goal scales and is not reported. Inspection of the MIs of the two-factor model showed that freeing the residual correlation between item 6 (Goal factor; “treatment goals both agreed upon”) and item 10 (Task factor; “work on problems in right way”) would improve model fit. The modified two-factor model had acceptable goodness-of-fit assessed by the CFI (0.965), SRMR (0.066) and the TLI (0.956), but not according to the RMSEA (0.101). To further improve model fit we inspected the MIs for the modified two-factor model and found that freeing the residual correlation between item 2 (Task factor; “insight in problems”) and item 8 (“Task factor; “agree what is important”) would improve model fit.

**Longitudinal measurement invariance.** At both time points the two-factor model consistently had the best fit for the WAI-S youth sample. As such, configural invariance was supported. To assess for scalar invariance a longitudinal MCFA model was set up for the two time points. Scalar invariance was supported by the criterions ∆CFI (= 0.000), ∆RMSEA (= 0.003), ∆SRMR (= 0.003) and ∆TLI (0.000) (see upper part of the supplementary Table S4 in the Appendix). A visual representation of the WAI-S youth modified longitudinal measurement invariance model is shown in figure S3 in the Appendix.

**Table 4:** Model Fit for WLSMV estimation in multilevel CFA of first-session and 2-month follow-up WAI-S patient and therapist sample

WAI-S sample	Model	χ2	df	CFI	RMSEA	SRMR	TLI
**Patients** First session (*n* = 203)							
	One-factor	238.205***	54	**0.918**	0.130	**0.079**	0.899
	Two-factor	187.738***	53	**0.940**	0.112	**0.068**	0.940
	Two-factor – modified with one residual correlation	144.964***	52	**0.958**	0.094	**0.060**	0.947
	Two-factor - modified with one residual correlation and one dual loading	127.685***	51	**0.966**	0.086	**0.055**	**0.956**
	Two-factor - modified with one residual correlation and two dual loadings	97.583***	50	**0.979**	**0.068**	**0.048**	**0.972**
2-month follow-up (*n* = 183)							
	One-factor	188.115***	54	**0.952**	0.116	**0.071**	0.941
	Two-factor	172.816***	53	**0.957**	0.111	**0.069**	0.947
	Two-factor – modified with one residual correlation	149.263***	52	**0.965**	0.101	**0.066**	**0.956**
	Two-factor – modified with two residual correlations	127.242***	51	**0.973**	0.090	**0.060**	**0.965**
**Therapists**							
First session (*n* = 203)							
	One-factor	193.467***	54	**0.946**	0.113	**0.078**	0.934
	Two-factor	150.240***	53	**0.962**	0.095	**0.064**	**0.953**
	Three-factor	137.332***	51	**0.966**	0.091	**0.060**	**0.957**
	Three-factor - modified with one dual loading	94.911***	50	**0.983**	**0.067**	**0.049**	**0.977**
2-month follow-up (*n* = 188)							
	One-factor One-factor – modified with one residual correlation One-factor – modified with two residual correlations One-factor – modified with three residual correlations	242.780*** 203.408*** 182.312*** 169.729***	54 53 52 51	**0.952** **0.962** **0.967** **0.970**	0.136 0.123 0.115 0.111	0.084 **0.075** **0.067** **0.060**	0.941 **0.952** **0.958** **0.961**
	Two-factor	240.983***	53	**0.952**	0.137	0.084	0.940
	Two-factor modified with one residual correlation	200.334***	52	**0.962**	0.123	**0.074**	**0.952**
	Two-factor – modified with two residual correlations	179.707***	51	**0.967**	0.116	**0.067**	**0.958**
	Two-factor – modified with three residual correlations	165.417***	50	**0.971**	0.111	**0.059**	**0.961**
	Three-factor	238.391***	51	**0.952**	0.140	0.082	0.938
	Three-factor – modified with one residual correlation	192.711***	50	**0.964**	0.123	**0.071**	**0.952**
	Three-factor – modified with one residual correlation and one dual loading	170.982***	49	**0.969**	0.115	**0.063**	**0.958**
	Three-factor – modified with two residual correlations and one dual loading	153.530***	48	**0.973**	0.108	**0.062**	**0.963**

Note. χ2 = Chi-square statistic; df = degrees of freedom; CFI = Comparative Fit Index (> .90); RMSEA = Root Mean Square Error of Approximation (< .08); SRMR = Standardized Root Mean Square Residual (< .08); TLI = Tucker-Lewis Index (> .95); **values in bold** meet the criterion for acceptable model fit; *** = *p* < .001. At both timepoints the three-factor model could not be reliably estimated and the bi-factor model could not be identified, these are not reported.

The two-factor model, modified with two residual correlations, had acceptable goodness-of-fit assessed by the CFI (0.973), SRMR (0.060) and the TLI (0.965), but the RMSEA (0.090) suggested a poor fit. The Bond and Collaboration factors in the modified two-factor model did not show a very large correlation, with a latent variable correlation of *r* = 0.61. A visual representation of the modified two-factor model at month two is shown in figure S2 in the Appendix.

**Factor structure WAI-S therapist.** The fit statistics of the three models tested for the WAI-S therapist are presented in the lower part of Table 4.

**First session.** As can be seen from Table 4, all three models did not fit well according to RMSEA, in addition, the one-factor model did not fit well according to the TLI. To improve model fit of the best fitting model we inspected the MI’s of the three-factor model and allowed item 11 to load also on the Task factor (MI = 37.2) The modified three-factor model revealed an acceptable fit according to the CFI (0.983), RMSEA (0.067), SRMR (0.049) and the TLI (0.977). The latent variable correlation between Task and Goal was *r* = 0.72 and the correlations between Bond with Task and Goal was *r* = 0.56 and *r* = 0.67, respectively. A visual representation of the WAI-S therapist modified three-factor model at first-session is shown in figure S4 in the Appendix.

**Two-months.** All three models did not fit well according to the RMSEA, SRMR and TLI (Table 4). Because the differences between the fit measures of the three models were small, we examined the MIs of all models. Regarding the one-factor model, we modified the model in three steps by freeing one residual correlation in each step (step 1) item 3 with item 6; (step 2) item 1 with item 6; (step 3) item 1 with item 3. As can be seen in Table 4, the one-factor model modified with three residual correlations had acceptable goodness-of-fit assessed by the CFI (0.970) and TLI (0.961) but a poor fit by RMSEA (0.111) and SRMR (0.060).

Regarding the two-factor model, we modified the model again in three steps by freeing one residual correlation in each step (step 1) item 3 with item 6; (step 2) item 1 with item 6; and (step 3) item 1 with item 3. The two-factor model modified with three residual correlations had acceptable goodness-of-fit assessed by the CFI (0.971), SRMR (0.059) and TLI (0.961) but a poor fit by the RMSEA (0.111). The latent variable correlation between Bond and Collaboration was moderate (*r* = 0.67).

Regarding the three-factor model, we modified the model in three steps by freeing in step 1 the residual correlation between item 3 and item 6, in step 2 to allow item 11 to load both on the Goal and Bond factors and in step 3 freeing the residual correlation between item 8 and item 9. The three-factor model, modified with two residual correlations and one dual loading, did not fit well according to the RMSEA (0.108) whereas the CFI (0.973), SRMR (0.062) and TLI (0.963) showed acceptable fit (Table 4). The latent variable correlation between Goal and Task was *r* = 0.83 and the correlations of Bond with Task and Goal were *r* = 0.60 and *r* = 0.68, respectively. Although the differences between the fit measures of the modified two- and three-factor models were small the three factor model had a slightly better fit, hence, a visual representation of the WAI-S therapist modified three-factor model at month two is shown in figure S5 in the Appendix.

**Longitudinal measurement invariance.** At both time points the three-factor model consistently had the best fit for the WAI-S therapist sample. As such, configural invariance was supported. When therapists’ ratings were compared on the three-factor model for both time points, scalar invariance was considered as lacking specifically due to TLI variation, ∆CFI (= 0.006), ∆RMSEA (= 0.003), ∆SRMR (= 0.000) and ∆TLI (0.013) (see lower part of the supplementary Table S4 in the Appendix).

### Nested data

Our data has a hierarchical structure in which youth are nested in therapists. Specifically, 7% of therapists treated one youth, 47% treated two-four youth and 46% treated five youth or more. The ICCs range was between 0.25 and 0.41 for the WAI-S youth and between 0.40 and 0.67 for the WAI-S therapist (see fourth line from the bottom in the Appendix, Tables S5–S8). Hence, for all items the dependency due to therapist was high or very high. Information about the fit statistics of the first session WAI-S models, without considering nesting within therapists or without considering the categorical nature of the data (i.e. using MLR estimation method), are displayed in Table S9 and Table S10 respectively in the supplementary material.

In our multilevel modeling we generally find lower chi-square values, lower RMSEA values, higher CFI values and higher TLI values by taking into account data clustering than when the hierarchical data structure was ignored. Although the changes in the fit statistics were in the favorable direction, this did not lead to a different factor structure.

## Discussion

Therapeutic alliance is considered to play an important role in youth treatment. Since the emphasis on the specific components of alliance may differ by treatment type, a good instrument to measure (the components of) alliance in youth treatment is relevant. However, most earlier psychometric studies in youth psychotherapy do not find support for the hypothesized three dimensional structure of the therapeutic alliance in youth. In this study, we addressed some methodological limitations of previous psychometric studies of the commonly used WAI-S questionnaire in youth by accounting for clustering in the data and by rephrasing two negatively worded items into positively worded items, to avoid possible response bias. The results from our multilevel confirmatory factor analyses provided partial support for Bordin’s [8] Task-Goal-Bond model of alliance and suggests a two-factor model for youth and a three-factor model for their adult therapist.

Concerning the youth’s perspective, the three-factor model could not be reliably estimated in our study at start of the treatment nor at two-months follow-up and the model could not be identified. The two-factor (collaboration and bond) model showed a better fit than the one-factor model at both time points. In addition, the measurement invariance analyses showed longitudinal measurement invariance for the two-factor model, suggesting that how youth interpret and rate the WAI-S does not change during the first months of treatment. Our findings differs from earlier studies on therapeutic alliance in youth, although Cirasola et al. [31] did find some support for the two-dimensional structure. However, although the two-factor youth alliance structure appeared robust over time not all fit indices of the 2-month WAI-S indicated a good fit and post hoc modification indices were added. Most modifications were related to residual correlations between goal and task items (both part of the collaboration factor). As we considered it plausible, that youth attribute ‘work together in determining treatment goals’ (item 4) and ‘work on treatment goals both agreed upon’ (item 6) to the sense of being understood by the therapist (bond), two dual loadings on the bond factor were added.

One explanation for the deviation of our findings from those of previous studies may lie in the versions of the WAI that have been used. The current study is the first factor analytic study investigating the WAI-S in which only positively worded items were used in youth treatment. In adult studies, in line with our findings, most evidence was found for the two dimensional structure of the patient’s alliance [12, 16, 51]. Moreover, the negatively worded items in the WAI(-S) used in previous youth alliance studies, may have resulted in response bias as demonstrated by Cirasola et al. [31]. Because the negatively worded items were part of the collaborative subscales of the WAI(-S), the response bias may have prevented the collaborative part of the alliance from being distinguished from the relational part. This may be particularly be the case because youth tend to view the alliance primarily as an affective construct [23]. It may be that youth, like adults, do distinguish between the relational and collaborative aspects of the therapeutic alliance but that this has not previously been demonstrated in youth alliance studies because previous WAI versions did not detect it. Our results show better psychometric properties of WAI-S relative to previous forms. However, the Chi-square values in all our final modified models did not indicate a good fit despite good fit indices within commonly accepted norms. This was previously demonstrated in adult research [7, 12, 16] and is possibly because the chi-square test is biased to be significant with large correlations between variables and/or skewed data [50, 52] which both are characteristics of alliance data. Accordingly, we found moderate correlations between Task and Goal and between Bond and Collaboration subscales on both time points, indicating that these dimensions are related but also distinguishable and may comprise different aspects of the alliance.

An alternative explanation for our two-dimensional alliance structure in youth psychotherapy may be the use of multi-level factor analyses in the present study. Because previous studies have not considered that youths are nested within therapists, their results may have been affected by biased parameter estimates, misestimated standard errors and model misfit. The majority of studies in youth provide no information about the number of youth per therapist [21, 29–31]. Therefore, model misfit due to the clustering of the data cannot be ruled out. This is an important consideration since our findings showed that not accounting for the clustering of youth within therapist yielded different fit measures compared to when the nested data structure was taken into account. Although these differences did not lead to different conclusions about the factor structure or can be tested for relevance, the changes were consistent and in the favorable direction. In addition, there was substantial dependency in the alliance data due to therapist as demonstrated by (very) large intraclass correlations in both samples.

Regarding the therapists’ perspective, the three-factor model seemed to represent the WAI-S structure at both time points. However, longitudinal measurement invariance was supported for configural invariance but not for scalar invariance. Since scalar invariance was not found, there could be a difference over time in how therapists interpret the scale. In addition, our results suggest that the three alliance dimensions become less distinguishable during treatment. Although the alliance structures appeared to be partially robust over time not all fit indices of the 2-month WAI-S indicated a good fit. These tentative confirmation of Bordin’s three dimensions of alliance for the therapists’ perspective does not support earlier studies reporting single [21, 29–31] or two [53] dimensions of alliance. However, we found moderate and strong correlations between Task and Goal subscales at first session and 2-months follow-up respectively. To be noted is that in the 2-months WAI-S model several post modification indices were added including a dual loading of item 11 on both the Goal and Bond factors. As it seems plausible that therapists may attribute ‘forming a clear understanding of the changes good for the youth’ to the agreement and collaboration around activities during therapy.

Since this is the first study in youth treatment that finds a best fit for a two-dimensional construct of alliance in youth, our findings still need to be interpreted with caution. Nevertheless, our study provides some possible implications for clinical practice and research, with regard to comparing alliance scores over time, across youths and therapists, between therapists and between youths. First, our study suggests that the concept of alliance in youth seems reasonably robust across the first months of treatment which allows comparisons across time of within-youth WAI-S Bond and Collaborative subscores during the first phase of treatment. For comparing within-therapist scores on the WAI-S subscales over time, caution is needed because full longitudinal measurement invariance was not found. However, therapeutic alliance is a dynamic construct and to test the robustness of this concept more assessments of alliance during treatment are needed. In addition, alliance is a dyadic construct in which both the youth and therapist are partners in co-creating the therapeutic relationship. In the present study the youth’ and therapist’ perspective on the alliance were related to each other at 2-months follow-up but not at the start of the treatment. Furthermore, in an earlier study, we found that evaluating the therapeutic relationship from both the youth and therapist perspective at the start of treatment is a stronger predictor of treatment outcome than using only one perspective in mental health and addiction care [54].

Second, the present study suggests a two-dimensional and three-dimensional concept of alliance for youth and therapist, respectively. Therefore, it could be argued that it might be best to use the total WAI-S score for the comparison of youth-rated and therapist-rated alliance scores. Third, when comparing alliance scores between therapists it is, given the especially strong intraclass correlations, important to realize that therapist alliance ratings are influenced by therapists rating styles. That is, therapists differ in their tendency to rate the level of alliance, irrespective of the patient. Caution is also needed in comparing the youth alliance scores between therapists. Because youth rate the alliance with only one therapist, it is not possible to assess the youth’s tendency to rate the alliance irrespective of the therapist. Furthermore, our findings suggest that youth’s in youth mental health or addiction care do distinguish between the relational and collaborative aspects of the alliance with their therapist. This may imply that youths and therapists may be able to concentrate on the goals and tasks of treatment even if they find it difficult to form a bond with each other or vice versa. Distinguishing these different components of alliance has implications for the clinical use of the WAI-S in youth mental health and addiction care, because most likely different types of treatments require different alliance `profiles` [8, 15, 18–20].

Our study has several limitations that should be mentioned. First, we had a relatively small therapist sample. However, in this relatively small sample we were able to control for therapist clustering, and this is the first psychometric study of youth-therapist alliance in youth treatment that applied multilevel confirmatory factor analysis. Nevertheless, future research with larger therapist samples is needed to replicate our findings. A second limitation could come from using MIs to improve model fit. Although using MIs is common practice, it should be noted that without using these modifications, none of the factor models demonstrated a good fit with our data. However, to limit the potential consequences of these modifications, the use of MIs was restricted and, each modification was based on both theoretical and statistical grounds.

## Conclusions

This study partly supports Bordin’s [8] Task-Goal-Bond model of alliance in mental health and addiction treatment of youth. From the therapists’ perspective the three-dimensional model seems most appropriate, while for youth a two-dimensional model seems applicable. This is the first psychometric study of the WAI-S in youth treatment in which only positively worded items were used to prevent response bias, and in which clustering in the alliance data was considered. In addition, the current study is the first to find a best fit for the two-dimensional construct of alliance in youth. If replicated in future studies, it could be that youth, like adults, do distinguish between the relational and collaborative aspects of the therapeutic alliance.

### Electronic supplementary material

Below is the link to the electronic supplementary material.

Supplementary Material 1

## Data Availability

The datasets analyzed during the current study are not publicly available due we did not request consent from study participants for using their coded (anonymous) study data for future research purposes but are available from the corresponding author on reasonable request.

## Abbreviations

WAI: Working Alliance Inventory (WAI)
WAI-S: Working Alliance Inventory Short
WAI-SR: Working Alliance Inventory Short Revised
MCFA: Multilevel Confirmatory Factor Analyses
PACT: Professional Alliance with Clients in Treatment
WLSMV: Weighted Least Squares Mean and Variance adjusted
MLR: Maximum Likelihood with Robust standard errors
RMSEA: Root Mean Square Error of Approximation
CFI: Comparative Fit Index
SRMR: Standardized Root Mean Square Residual
MI: Modification Index
ICC: IntraClass Correlations

## References

[1] Flückiger C, Del Re AC, Wampold BE, Horvath AO (2018). The alliance in adult psychotherapy: a meta-analytic synthesis. Psychother (Chicago III).

[2] Karver M, De Nadai AS, Monahan M, Shirk SR (2018). Meta-analysis of the prospective relation between alliance and outcome in child and adolescent psychotherapy. Psychother (Chicago III).

[3] Murphy R, Hutton P, Practitioner, Review (2018). Therapist variability, patient-reported therapeutic alliance, and clinical outcomes in adolescents undergoing mental health treatment - a systematic review and meta-analysis. J Child Psychol Psychiatry.

[4] Horvath G (1989). Development and validation of the Working Alliance Inventory. J Couns Psychol.

[5] Paap D, Karel YH, Verhagen AP, Dijkstra PU, Geertzen JH, Pool G. The Working Alliance Inventory’s Measurement Properties: A Systematic Review. Front Psychol. 2022;13. 10.3389/fpsyg.2022.945294 PMC9337219 35910993

[6] Tracey TJ, Kokotovic AM (1989). Factor structure of the working alliance inventory. Psychol Assessment: J Consulting Clin Psychol.

[7] Hatcher RL, Gillaspy JA (2006). Development and validation of a revised short version of the Working Alliance Inventory. Psychother Res.

[8] Bordin ES (1979). The generalizability of the psychoanalytic concept of the working alliance. Psychotherapy: Theory Res Pract.

[9] Bordin ES (1980). Counseling psychology in the year 2000: prophecy of wish fulfillment?. Couns Psychol.

[10] Martin DJ, Garske JP, Davis MK (2000). Relation of the therapeutic alliance with outcome and other variables: a meta-analytic review. J Consult Clin Psychol.

[11] Busseri MA, Tyler JD (2003). Interchangeability of the working alliance inventory and working alliance inventory, short form. Psychol Assess.

[12] Munder T, Wilmers F, Leonhart R, Linster HW, Barth J (2010). Working Alliance Inventory-Short revised (WAI‐SR): psychometric properties in outpatients and inpatients. Clin Psychol Psychotherapy: Int J Theory Pract.

[13] Andrusyna TP, Tang TZ, DeRubeis RJ, Luborsky L (2001). The factor structure of the Working Alliance Inventory in cognitive-behavioral therapy. J Psychother Pract Res.

[14] Hatcher RL, Barends AW (1996). Patients’ view of the alliance in psychotherapy: exploratory factor analysis of three alliance measures. J Consult Clin Psychol.

[15] Webb CA, DeRubeis RJ, Amsterdam JD, Shelton RC, Hollon SD, Dimidjian S (2011). Two aspects of the therapeutic alliance: differential relations with depressive symptom change. J Consult Clin Psychol.

[16] Falkenström F, Hatcher RL, Holmqvist R (2015). Confirmatory factor analysis of the patient version of the Working Alliance inventory–short form revised. Assessment.

[17] Smits D, Luyckx K, Smits D, Stinckens N, Claes L (2015). Structural characteristics and external correlates of the Working Alliance Inventory-Short Form. Psychol Assess.

[18] Castonguay, Constantino, Holtforth (2006). The working alliance: where are we and where should we go?. Psychother Theory Res Pract Train.

[19] Muran JC, Barber JP. The therapeutic alliance: an evidence-based guide to practice. Guilford Press; 2011.

[20] Raue PJ, Goldfried MR. Cognitive-Behavior Therapy. The working alliance: Theory, research, and practice. 1994;173:131.

[21] DiGiuseppe R, Linscott J, Jilton R (1996). Developing the therapeutic alliance in child—adolescent psychotherapy. Appl Prev Psychol.

[22] Figueiredo B, Dias P, Lima VS, Lamela D. Working alliance inventory for children and adolescents (WAI-CA). Eur J Psychol Assess. 2016.

[23] Ormhaug SM, Shirk S, Wentzel-Larsen T (2015). Therapist and client perspectives on the alliance in the treatment of traumatized adolescents. Eur J Psychotraumatology.

[24] Shirk SR, Karver M (2003). Prediction of treatment outcome from relationship variables in child and adolescent therapy: a meta-analytic review. J Consult Clin Psychol.

[25] Zack SE, Castonguay LG, Boswell JF (2007). Youth working alliance: a core clinical construct in need of empirical maturity. Harv Rev Psychiatry.

[26] Gulliver A, Griffiths KM, Christensen H (2010). Perceived barriers and facilitators to mental health help-seeking in young people: a systematic review. BMC Psychiatry.

[27] Block AM, Greeno CG (2011). Examining outpatient treatment dropout in adolescents: a literature review. Child Adolesc Soc Work J.

[28] De Haan AM, Boon AE, de Jong JT, Hoeve M, Vermeiren RR (2013). A meta-analytic review on treatment dropout in child and adolescent outpatient mental health care. Clin Psychol Rev.

[29] Diamond G, Liddle HA, Wintersteen MB, Dennis ML, Godley SH, Tims F (2006). Early therapeutic alliance as a predictor of treatment outcome for adolescent cannabis users in outpatient treatment. Am J Addictions.

[30] Anderson RE, Spence SH, Donovan CL, March S, Prosser S, Kenardy J (2012). Working alliance in online cognitive behavior therapy for anxiety disorders in youth: comparison with clinic delivery and its role in predicting outcome. J Med Internet Res.

[31] Cirasola A, Midgley N, Fonagy P, Consortium I, Martin P (2021). The factor structure of the Working Alliance Inventory short-form in youth psychotherapy: an empirical investigation. Psychother Res.

[32] Julian MW (2001). The consequences of ignoring multilevel data structures in nonhierarchical covariance modeling. Struct Equ Model.

[33] Kaplan D, Elliott PR (1997). A didactic example of multilevel structural equation modeling applicable to the study of organizations. Struct Equation Modeling: Multidisciplinary J.

[34] Muthen BO, Satorra A. Complex sample data in structural equation modeling. Sociol Methodol. 1995:267–316.

[35] Pornprasertmanit S, Lee J, Preacher KJ (2014). Ignoring clustering in confirmatory factor analysis: some consequences for model fit and standardized parameter estimates. Multivar Behav Res.

[36] Hox JJ, Moerbeek M, Van de Schoot R. Multilevel analysis: Techniques and applications: Routledge; 2017.

[37] van Benthem P, Spijkerman R, Blanken P, Kleinjan M, Vermeiren RRJM, Hendriks VM (2020). A dual perspective on first-session therapeutic alliance: strong predictor of youth mental health and addiction treatment outcome. Eur Child Adolesc Psychiatry.

[38] Stinckens N, Ulburghs A, Claes L (2009). De Werkalliantie als sleutelelement in het therapiegebeuren. Meting met behulp Van De WAV-12: de Nederlandse vertaling Van De Working Alliance. Inventory Tijdschr Klin Psychol.

[39] Brown TA. Confirmatory factor analysis for applied research. Guilford; 2015.

[40] Kline RB. Principles and practice of structural equation modeling. Guilford; 2015.

[41] Yu C-Y. Evaluating cutoff criteria of model fit indices for latent variable models with binary and continuous outcomes. University of California, Los Angeles; 2002.

[42] Lt H, Bentler PM (1999). Cutoff criteria for fit indexes in covariance structure analysis: conventional criteria versus new alternatives. Struct Equation Modeling: Multidisciplinary J.

[43] West SG, Taylor AB, Wu W (2012). Model fit and model selection in structural equation modeling. Handb Struct Equation Model.

[44] Millsap RE. Statistical approaches to measurement invariance. Routledge; 2012.

[45] Chen FF (2007). Sensitivity of goodness of fit indexes to lack of measurement invariance. Struct Equation Modeling: Multidisciplinary J.

[46] Gliem JA, Gliem RR, editors. Calculating, interpreting, and reporting Cronbach’s alpha reliability coefficient for Likert-type scales2003: Midwest Research-to-Practice Conference in Adult, Continuing, and Community &#8230.

[47] Musca SC, Kamiejski R, Nugier A, Méot A, Er-Rafiy A, Brauer M (2011). Data with hierarchical structure: impact of intraclass correlation and sample size on type-I error. Front Psychol.

[48] Twisk JW. Applied multilevel analysis: a practical guide for medical researchers. Cambridge University Press; 2006.

[49] Muthén LK, Muthén B. Mplus user’s guide: Statistical analysis with latent variables, user’s guide. Muthén & Muthén; 2017.

[50] Kenny DA. Measuring model fit. 2015.

[51] Ross EC, Polaschek DL, Wilson M (2011). Shifting perspectives: a confirmatory factor analysis of the working alliance inventory (short form) with high-risk violent offenders. Int J Offender Ther Comp Criminol.

[52] Alavi M, Visentin DC, Thapa DK, Hunt GE, Watson R, Cleary ML. Chi-square for model fit in confirmatory factor analysis. 2020. 10.1111/jan.14399 32323338

[53] Hatcher RL, Lindqvist K, Falkenström F (2020). Psychometric evaluation of the Working Alliance Inventory—Therapist version: current and new short forms. Psychother Res.

[54] van Benthem P, Spijkerman R, Blanken P, Kleinjan M, Vermeiren RR, Hendriks VM (2020). A dual perspective on first-session therapeutic alliance: strong predictor of youth mental health and addiction treatment outcome. Eur Child Adolesc Psychiatry.

