# Supplementary material for: The working alliance inventory – short version: psychometric properties of the patient and therapist form in youth mental health and addiction care
Source: BMC Psychol. 2024 May 31;12:319. doi: 10.1186/s40359-024-01754-1 (PMC11143678; doi:10.1186/s40359-024-01754-1)

Appendix

Tables

Table S1. Participant characteristics at baseline for 2-month follow-up completers and non-completers

	2-month follow-up completers (n = 183) Mean (s.d.)	2-month follow-up non-completers (n = 20) Mean (s.d.)	t-test / M-W *p*
**Youth Addiction Care**	n = 81	n = 15	
Days primary substance use/ problem behavior past month[a]	12.7 (12.0)	17.3 (14.2)	.096[b]
**Youth Mental Health Care**	n = 102	n = 5	
Strength and Difficulties Questionnaire (SDQ score: 0-40)	15.2 (5.2)	19.0 (3.3)	.107[c]
**First session Therapeutic Alliance**	n = 102	n = 20	
Youth-rated WAI-S (1-5)	3.9 (0.6)	4.1 (0.4)	.852[b]
Therapist-rated WAI-S (1-5)	4.0 (0.5)	3.8 (0.6)	.467[b]

[a] Measured with the MATE-Y; [b] Using Mann-Whitney test; [c] Using t-test;
MATE-Y: measurement in the Addiction for Triage and Evaluations, Youth version; SDQ: Strength and Difficulties Questionnaire; WAI-S: Working Alliance Inventory-short version.

Table S2. Means and standard deviations of the 2-month follow-up WAI-S items patient and therapist version

		mean(s.d.)		mean(s.d.)
WAI-S	Patient version		Therapist version	
		**Task**		
**Item 2**	What I do in this treatment gives me more insight into my problems	3.5 (1.0)	The youth and I have confidence in the usefulness or our current activities in the treatment	3.5 (0.9)
**Item 8**	The therapist and I agree about what is important for me to work on	4.1 (0.9)	The youth and I agree about what is important to work on	3.7 (0.8)
**Item 10**	I think that my contribution to this treatment will help me to achieve the changes I want	3.8 (1.1)	I am confident that the things we do in treatment will help the youth to achieve the changes he/she wanted	3.7 (0.8)
**Item 12**	I believe that the way we work on the problems is the right way	3.7 (1.1)	The youth believe(s) that the way we work on his/her problems is the right way	3.4 (1.0)
		**Goal**		
**Item 1**	One result of this treatment is that it is clearer for me how I can change	3.4 (1.1)	One result of this treatment is that it is clearer for the youth how he/she can change	3.6 (0.9)
**Item 4**	The therapist and I work together in determining the treatment goals	4.3 (0.9)	The youth and I worked together to determine treatment goals	4.0 (0.9)
**Item 6**	The therapist and I work on treatment goals we both agreed upon	4.3 (0.9)	The youth and I work on treatment goals we both agreed upon	4.1 (0.9)
**Item 11**	The therapist and I have formed a clear understanding of the kind of changes that would be good for me	3.8 (1.1)	The youth and I have formed a good understanding of the kind of changes that would be good for him/her	3.6 (0.9)
		**Bond**		
**Item 3**	I believe the therapist likes me	4.4 (0.8)	I believe that the youth likes me	3.8 (0.8)
**Item 5**	The therapist and I respect each other	4.8 (0.5)	The youth and I respect each other	4.5 (0.7)
**Item 7**	I feel appreciated by the therapist	4.4 (0.8)	I appreciate the youth as a person	4.5 (0.6)
**Item 9**	I feel the therapist cares for me, even if I do things he/she disapproves of	4.0 (1.1)	I respect the youth, even if he/she does things I don't approve of	4.5 (0.6)

Table S3. Correlations of first-session and 2-month follow-up WAI-S factors in patient and therapist version

**WAI-S therapist**	**WAI-S patient**			
	Task	Goal	Bond	Collaboration
First-session				
Task	-0.06	-0.03	-0.03	-0.06
Goal	-0.05	-0.02	-0.02	-0.04
Bond	-0.05	-0.00	0.01	0.03
Collaboration	-0.07	-0.03	-0.02	-0.06
2-month follow-up				
Task	0.31**	0.32**	0.22**	0.34**
Goal	0.30**	0.28**	0.23**	0.31**
Bond	0.12	0.12	0.19**	0.13
Collaboration	0.32**	0.32**	0.24**	0.34**

Table S4. Longitudinal measurement invariance (configural and scalar) for WAI-S patient and therapist version

WAI-S sample	Model	χ2	df	CFI	RMSEA	SRMR	TLI	Model comparison	Δχ2	ΔCFI	ΔRMSEA	ΔSRMR	ΔTLI
**Patients**													
	Configural	394.369***	288	**0.971**	**0.043**	0.082	**0.971**						
	Scalar	430.495***	326	**0.971**	**0.040**	**0.079**	**0.971**	Scalar vs Configural	36.126	**0.000**	**0.003**	**0.003**	**0.000**
**Therapists**													
	Configural	908.725***	283	0.806	0.104	0.345	0.778						
	Scalar	957.931***	311	0.800	0.101	0.345	0.791	Scalar vs Configural	49.206	**0.006**	**0.003**	**0.000**	0.013

Note. Model fit: χ2 = Chi-square statistic; df = degrees of freedom; CFI = Comparative Fit Index (> .90); RMSEA = Root Mean Square Error of Approximation (<.08); SRMR = Standardized Root Mean Square Residual (<.08); TLI = Tucker-Lewis Index (> .95); **values in bold** meet the criterion for acceptable model fit; *** = $p < .001$. Model comparison: Models were compared based on Chen's (2007) criteria: changes in CFI (Δ < 0.01), RMSEA (Δ < 0.015), SRMR (Δ < 0.02) and TLI (Δ < 0.01); **values in bold** indicate non-significant model change.

Table S5. Correlation matrix and descriptive statistics of first-session WAI-S youth (n=203)

	Item1	Item2	Item3	Item4	Item5	Item6	Item7	Item8	Item9	Item10	Item11	Item12
Item1	1											
Item2	0.53	1										
Item3	0.37	0.33	1									
Item4	0.36	0.34	0.43	1								
Item5	0.21	0.14	0.45	0.47	1							
Item6	0.27	0.23	0.45	0.53	0.43	1						
Item7	0.24	0.22	0.64	0.51	0.46	0.59	1					
Item8	0.24	0.24	0.43	0.40	0.31	0.44	0.39	1				
Item9	0.27	0.18	0.46	0.35	0.31	0.31	0.52	0.37	1			
Item10	0.40	0.45	0.37	0.44	0.32	0.39	0.41	0.41	0.29	1		
Item11	0.35	0.40	0.27	0.48	0.28	0.35	0.32	0.47	0.40	0.53	1	
Item12	0.35	0.38	0.31	0.33	0.26	0.30	0.32	0.43	0.37	0.51	0.63	1
ICC	0.35	0.35	0.34	0.25	0.28	0.30	0.29	0.29	0.35	0.31	0.38	0.28
Skewness	-0.08	-0.21	-0.97	-0.99	-1.79	-1.19	-1.29	-0.85	-0.54	-0.52	-0.61	-0.41
Excess kurtosis	-0.58	-0.77	0.93	0.72	2.31	0.90	1.57	0.54	-0.29	-0.35	-0.14	-0.55
N Missing	0	1	0	0	1	0	0	0	2	0	1	0

Table S6. Correlation matrix and descriptive statistics of first session WAI-S therapist (n=203)

	Item1	Item2	Item3	Item4	Item5	Item6	Item7	Item8	Item9	Item10	Item11	Item12
Item1	1											
Item2	0.37	1										
Item3	0.32	0.49	1									
Item4	0.25	0.46	0.49	1								
Item5	0.29	0.40	0.51	0.57	1							
Item6	0.32	0.46	0.38	0.64	0.62	1						
Item7	0.27	0.43	0.40	0.49	0.67	0.57	1					
Item8	0.29	0.56	0.38	0.46	0.43	0.60	0.43	1				
Item9	0.17	0.33	0.40	0.36	0.59	0.42	0.64	0.39	1			
Item10	0.24	0.55	0.33	0.30	0.28	0.36	0.42	0.38	0.27	1		
Item11	0.36	0.56	0.39	0.50	0.43	0.46	0.40	0.64	0.26	0.49	1	
Item12	0.33	0.58	0.30	0.33	0.32	0.40	0.29	0.58	0.24	0.43	0.65	1
ICC	0.55	0.47	0.40	0.57	0.60	0.59	0.63	0.46	0.67	0.50	0.42	0.41
Skewness	-0.34	-0.10	0.08	-0.82	-1.07	-0.62	-1.24	-0.20	-0.96	-0.42	-0.57	-0.30
Excess kurtosis	0.02	-0.45	-0.38	0.51	0.81	-0.35	1.11	-0.21	0.56	0.25	0.38	0.03
N Missing	1	0	0	0	0	0	0	0	1	0	0	2

Table S7. Correlation matrix and descriptive statistics of 2-month follow-up WAI-S youth (n=183)

	Item1	Item2	Item3	Item4	Item5	Item6	Item7	Item8	Item9	Item10	Item11	Item12
Item1	1											
Item2	0.50	1										
Item3	0.33	0.31	1									
Item4	0.42	0.31	0.53	1								
Item5	0.13	0.11	0.49	0.38	1							
Item6	0.34	0.31	0.52	0.63	0.32	1						
Item7	0.22	0.21	0.69	0.47	0.46	0.47	1					
Item8	0.43	0.39	0.54	0.48	0.32	0.47	0.48	1				
Item9	0.17	0.22	0.47	0.28	0.30	0.32	0.48	0.44	1			
Item10	0.42	0.33	0.44	0.47	0.23	0.48	0.40	0.53	0.39	1		
Item11	0.49	0.37	0.47	0.54	0.29	0.61	0.34	0.57	0.32	0.65	1	
Item12	0.42	0.42	0.41	0.38	0.21	0.52	0.44	0.60	0.40	0.54	0.64	1
ICC	0.36	0.41	0.37	0.29	0.28	0.31	0.34	0.35	0.38	0.28	0.39	0.41
Skewness	-0.27	-0.50	-1.09	-1.17	-2.40	-1.24	-1.24	-1.03	-0.95	-0.56	-0.70	-0.56
Excess kurtosis	-0.66	-0.49	0.53	0.70	5.21	1.08	0.77	0.82	0.36	-0.55	-0.24	-0.57
N Missing	0	1	0	0	0	1	0	0	1	0	0	0

Table S8. Correlation matrix and descriptive statistics of 2-month follow-up WAI-S therapist (n=188)

	Item1	Item2	Item3	Item4	Item5	Item6	Item7	Item8	Item9	Item10	Item11	Item12
Item1	1											
Item2	0.58	1										
Item3	0.34	0.56	1									
Item4	0.35	0.61	0.58	1								
Item5	0.25	0.43	0.58	0.59	1							
Item6	0.37	0.63	0.57	0.69	0.66	1						
Item7	0.25	0.38	0.49	0.52	0.68	0.59	1					
Item8	0.45	0.68	0.51	0.59	0.50	0.66	0.50	1				
Item9	0.13	0.32	0.44	0.36	0.56	0.47	0.66	0.40	1			
Item10	0.37	0.61	0.51	0.46	0.32	0.44	0.38	0.54	0.35	1		
Item11	0.42	0.64	0.52	0.63	0.42	0.58	0.41	0.70	0.35	0.59	1	
Item12	0.47	0.77	0.55	0.58	0.43	0.58	0.36	0.70	0.27	0.57	0.71	1
ICC	0.44	0.43	0.44	0.51	0.56	0.50	0.59	0.54	0.63	0.58	0.45	0.50
Skewness	-0.83	-0.37	-0.36	-0.91	-1.11	-0.99	-1.06	-0.52	-0.75	-0.62	-0.69	-0.38
Excess kurtosis	0.71	-0.16	0.01	0.64	0.45	0.56	0.05	0.08	-0.46	0.48	0.20	-0.42
N Missing	1	1	0	0	0	0	0	0	0	1	0	0

Table S9. Model Fit for WLSMV estimation in single level CFA of first-session WAI-S youth and therapist sample

	χ2	df	CFI	RMSEA	SRMR	TLI
**WAI-S youth sample**						
One-factor	267.562***	54	0.899	0.140	**0.078**	0.876
Two-factor	203.189***	53	**0.929**	0.118	**0.067**	0.911
Two-factor – modified with one residual correlation	161.410***	52	**0.948**	0.102	**0.060**	0.934
Two-factor - modified with one residual correlation and one dual loading	142.959***	51	**0.956**	0.094	**0.055**	0.944
Two-factor - modified with one residual correlation and two dual loadings	108.260***	50	**0.972**	**0.076**	**0.048**	**0.964**
**WAI-S therapist sample**						
One-factor	250.500***	54	**0.942**	0.134	**0.079**	0.929
Two-factor	184.855***	53	**0.961**	0.111	**0.065**	**0.951**
Three-factor	167.446***	51	**0.966**	0.106	**0.060**	**0.955**
Three-factor - modified with one dual loading	115.713***	50	**0.981**	**0.080**	**0.049**	**0.974**

Note. WLSMV= mean and variance adjusted weighted least squares; χ2 = Chi-square statistic; df = degrees of freedom; CFI = Comparative Fit Index (> .90); RMSEA = Root Mean Square Error of Approximation (<.08); SRMR = Standardized Root Mean Square Residual (<.08); TLI = Tucker-Lewis Index (> .95); **values in bold** meet the criterium for acceptable model fit; *** = p < .001. The three-factor model could not be reliably estimated in the youth sample at first session and is not reported.

Table S10. Model Fit for MLR estimation in multilevel CFA of first-session WAI-S youth and therapist sample

	χ2	df	CFI	RMSEA	SRMR	TLI
**WAI-S youth sample**						
One-factor	213.678***	54	0.792	0.122	**0.075**	0.746
Two-factor	163.049***	53	0.860	0.101	**0.068**	0.826
Two-factor – modified with one residual correlation	138.305***	52	0.890	0.090	**0.061**	0.861
Two-factor - modified with one residual correlation and one dual loading	117.195***	51	**0.916**	**0.080**	**0.058**	0.891
Two-factor - modified with one residual correlation and two dual loadings	96.250***	50	**0.941**	**0.068**	**0.052**	0.922
**WAI-S therapist sample**						
One-factor	259.352***	54	0.796	0.137	**0.076**	0.751
Two-factor	176.895***	53	0.877	0.107	**0.069**	0.847
Three-factor	162.563***	51	0.889	0.104	**0.063**	0.857
Three-factor - modified with one dual loading	119.656***	50	**0.931**	0.083	**0.056**	0.909

Note. MLR = maximum likelihood with robust standard errors; χ2 = Chi-square statistic; df = degrees of freedom; CFI = Comparative Fit Index (> .90); RMSEA = Root Mean Square Error of Approximation (<.08); SRMR = Standardized Root Mean Square Residual (<.08); TLI = Tucker-Lewis Index (> .95); **values in bold** meet the criterium for acceptable model fit; *** = $p < .001$. The three-factor model could not be reliably estimated in the youth sample at first session and is not reported.

Figure S1. WAI-S youth modified two-factor model first session

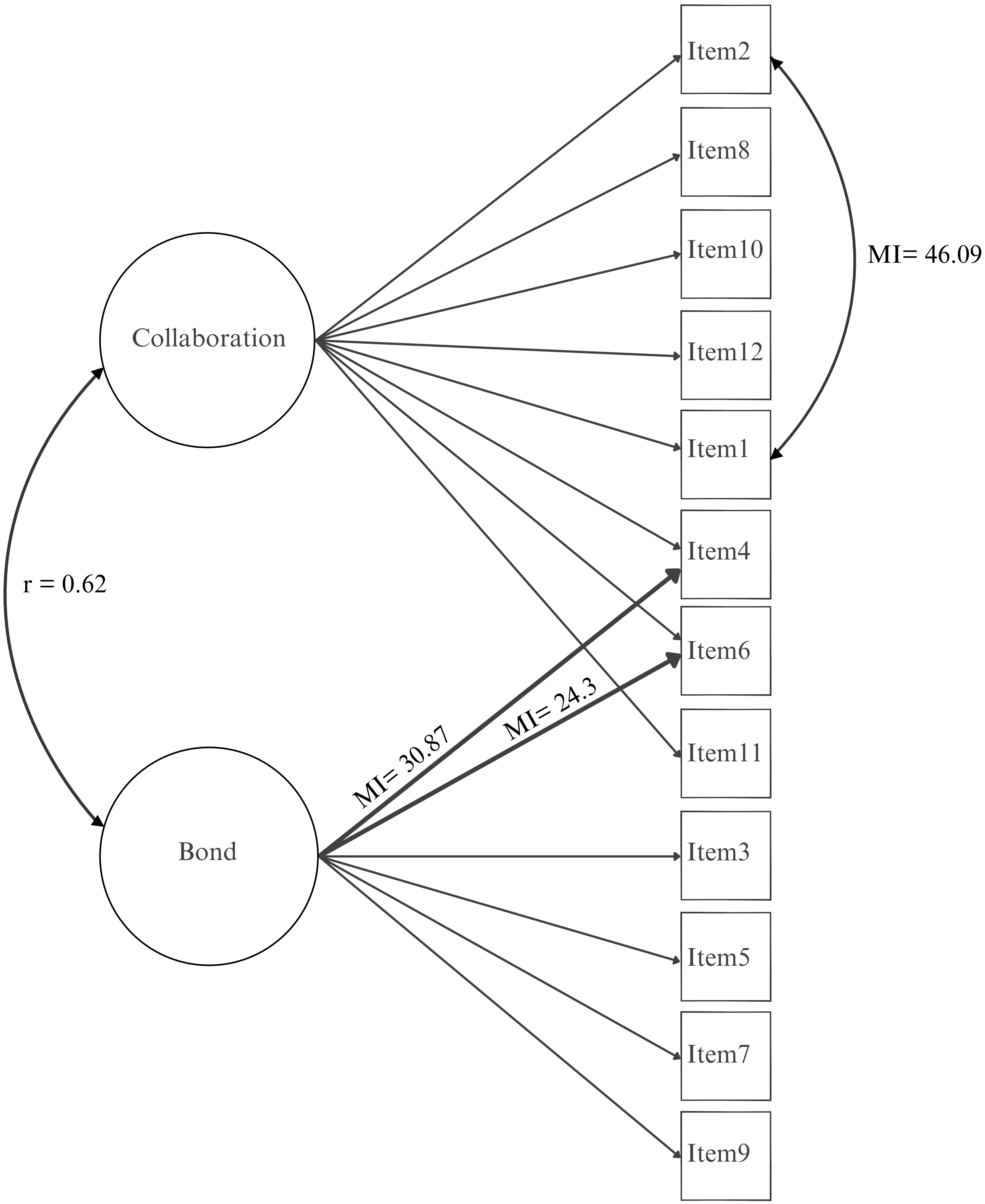

Figure S2. WAI-S youth modified two-factor model 2-months

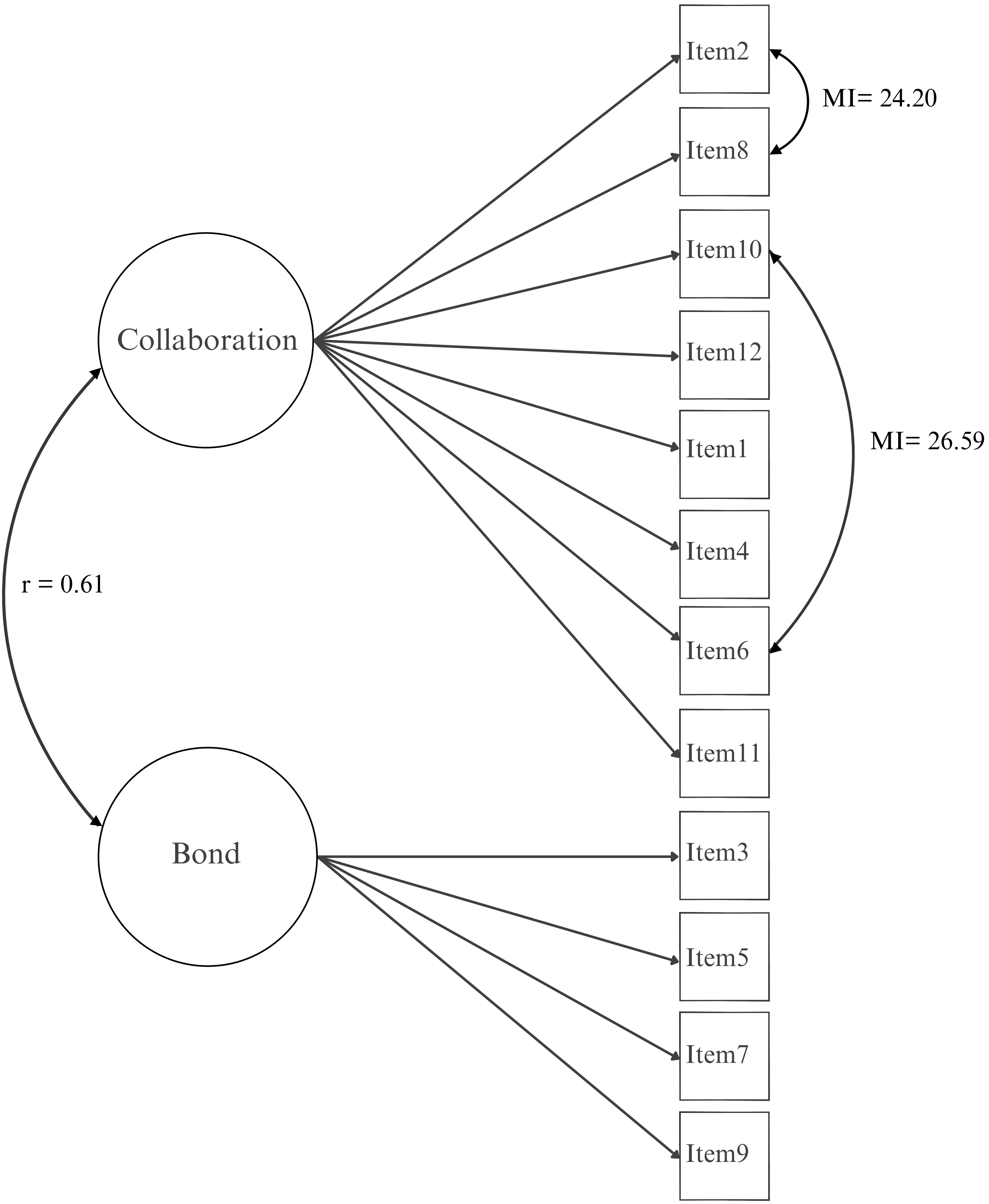

Figure S3. WAI-S youth modified longitudinal measurement invariance model

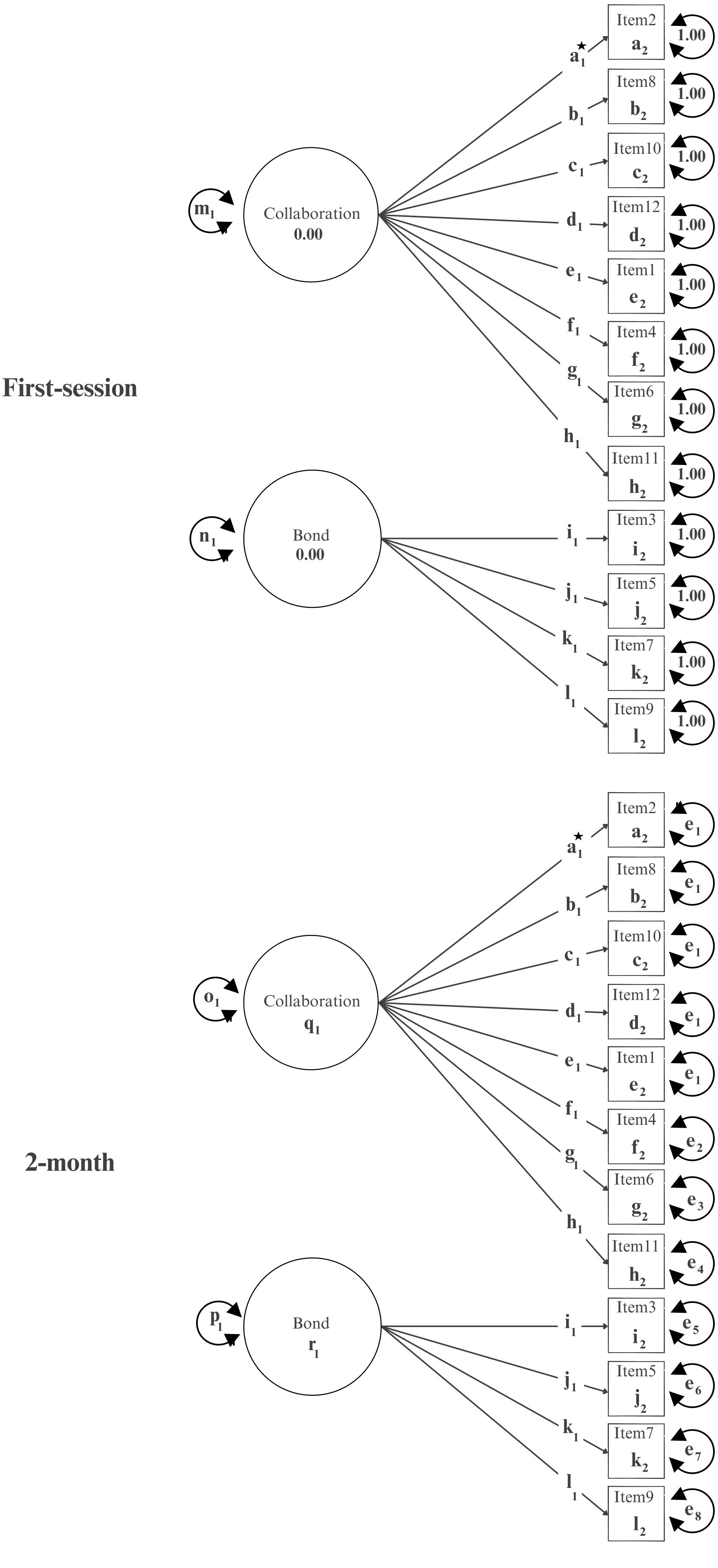

Note. In measurement invariance tests the models are fit to the first-session and 2-month follow-up group and parameters are constrained to be equal across the groups. Both factor loadings and item intercepts constrained to be equivalent across groups. ⋆ to identify the factor, this factor loading is set equal to 1.00.

Figure S4. WAI-S therapist modified three-factor model first session

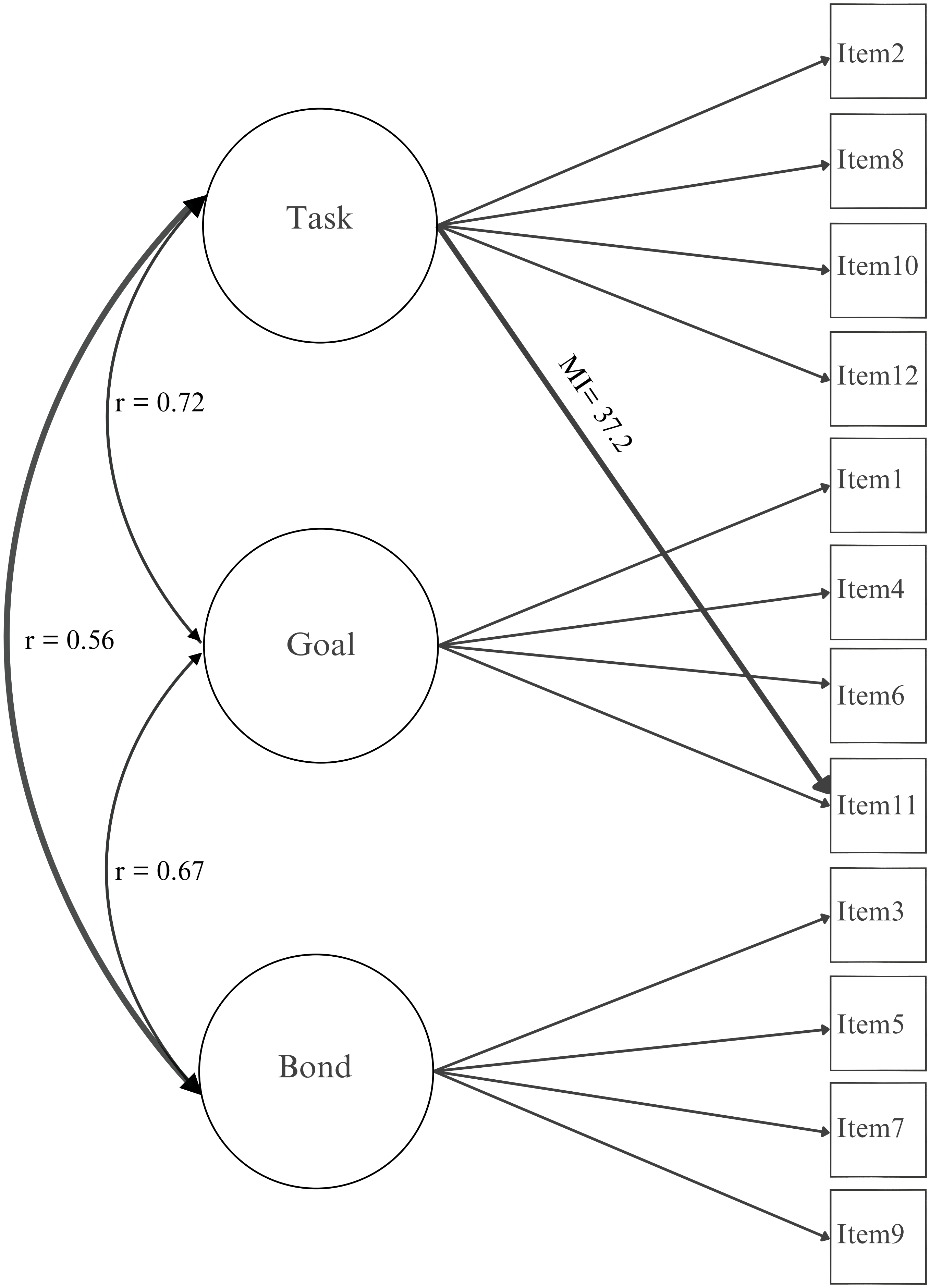

Figure S5. WAI-S therapist modified three-factor model 2-months

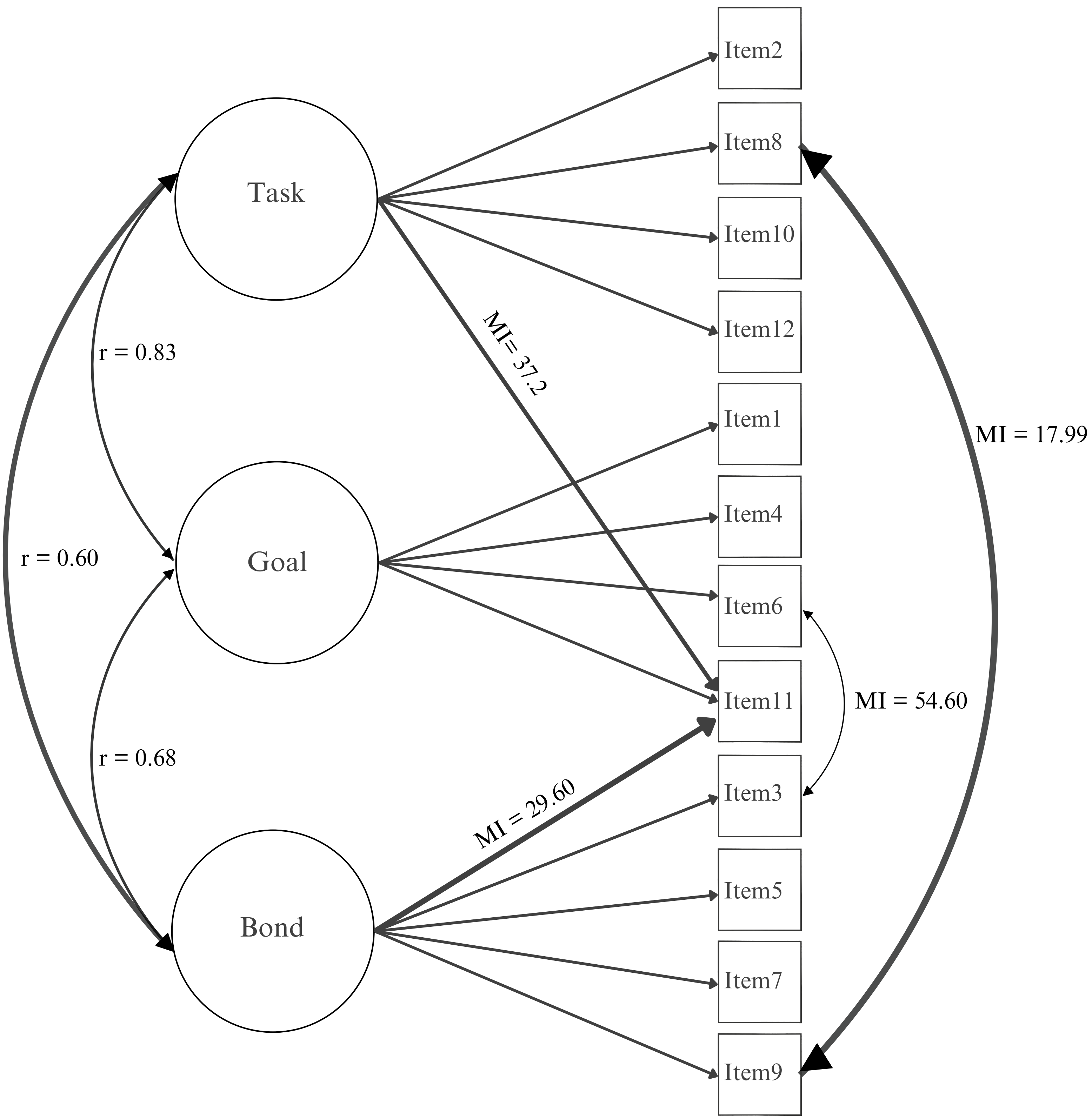

Supplement: Supplementary file 1 — Supplementary Material 1 [file 40359_2024_1754_MOESM1_ESM.pdf]
